# Evaluation of the Clinical Nursing Effects of a Traditional Chinese Medicine Nursing Program Based on Care Pathways for Patients With Type 2 Diabetes: Protocol for a Randomized Controlled Clinical Trial

**DOI:** 10.2196/58951

**Published:** 2025-03-31

**Authors:** Yanchun Zhao, Ting Huang, Yanli Chen, Songmei Li, Juan Zhao, Xu Han, Qing Ni, Ning Su

**Affiliations:** 1 Department of Endocrinology Guang'anmen Hospital China Academy of Chinese Medical Sciences Beijing China

**Keywords:** type 2 diabetes, traditional Chinese medicine, TCM nursing program, clinical pathway, application research, diabetes, diabetes mellitus, research protocol, nursing, nursing program, nursing care, chronic disease, disease monitoring, prevalence, China, adult, patient recovery, psychological care, health education, quality of life, blood glucose, self-care, medication, control group, patient satisfaction

## Abstract

**Background:**

To improve the performance of health care institutions, reduce overmedication, and minimize the waste of medical resources, China is committed to implementing a clinical pathway management model. This study aims to standardize nursing practices, foster clinical thinking in nurses, and promote patient recovery.

**Objective:**

The purpose of this study is to evaluate the clinical effects of a traditional Chinese medicine (TCM) nursing program based on nursing pathways for patients with type 2 diabetes mellitus (T2DM).

**Methods:**

This study uses a prospective, randomized, single-blind, parallel-controlled design. Based on sample size calculations, the study will include 594 patients with diabetes, with 2 groups of 297 patients: an observation group will receive a TCM nursing program based on clinical pathways, while a control group will receive routine care. Both groups will be evaluated before and after the intervention using assessment indicators. The primary outcome is the quality of life score, measured by a diabetes-specific quality of life questionnaire. Secondary outcomes include hospital stay duration, medical expenses, health knowledge, blood glucose control, symptom scores, and patient satisfaction.

**Results:**

This study was funded in August 2021 and has received approval from the Ethics Committee of Guang’anmen Hospital, China Academy of Chinese Medical Sciences (2022-022-KY-01). The trial is ongoing, with the first patient enrolled in September 2022. The study is expected to conclude in April 2025. To date, 380 patients have been recruited, with 202 randomized into the study, though no statistical analysis of the data has yet been conducted. A single-blind method is used; nurses are aware of group assignments and intervention plans, while patients remain blinded. Final results are planned for release in the first quarter of 2025.

**Conclusions:**

This study seeks to integrate existing national standardized nursing protocols with clinical pathways to implement more efficient and higher-quality nursing practices. The goal is to standardize nursing procedures, enhance patients’ quality of life, and improve self-care and medication adherence after discharge.

**Trial Registration:**

International Traditional Medicine Clinical Trial Registry ITMCTR2022000048; https://tinyurl.com/y4jd68h4

**International Registered Report Identifier (IRRID):**

DERR1-10.2196/58951

## Introduction

According to the Guideline for the Prevention and Treatment of Type 2 Diabetes Mellitus in China (2020 Edition) [1], the prevalence of diabetes in China has surged from 0.67% in 1980 to 11.2% in 2015-2017 [2,3]. Diabetes management in China faces significant challenges, particularly in rural areas where awareness, treatment, and control rates remain low. Large-scale epidemiological surveys conducted in 2010 and 2013 reported diabetes awareness rates of 30.1% and 36.5%, treatment rates of 25.8% and 32.2%, and control rates of 39.7% and 49.2%, respectively [3-5].

Most existing studies have focused on evaluating clinical nursing pathways or nursing programs. For instance, a study by Li [6] analyzed the effectiveness of clinical nursing pathways in type 2 diabetes mellitus (T2DM) care, demonstrating that a research group using clinical nursing pathways achieved better control of fasting blood glucose and postprandial 2-hour glucose levels compared to a control group (*P*<.05). The research group also had significantly fewer instances of improper hypoglycemia treatment, insulin injection errors, dietary mistakes, inadequate exercise, and nonadherence to medical advice (*P*<.05). Additionally, compared to the control group, the research group had shorter hospital stays, lower hospitalization costs, and higher overall nursing satisfaction (*P*<.05), indicating that the application of clinical nursing pathways can effectively control blood glucose levels, improve nursing quality, and enhance patient satisfaction.

Similarly, a study by Ren et al [7] showed that implementing traditional Chinese medicine (TCM) nursing programs in general hospitals resulted in higher quality-of-life scores for patients in an observation group 1 month after discharge compared to a control group. At discharge, the observation group had a significantly higher nursing satisfaction rate (93%) than the control group (77%; *P*<.05). This suggests that the TCM nursing program model can improve both quality of life and nursing satisfaction. These studies demonstrate that clinical nursing pathways or TCM nursing programs can effectively control blood glucose levels and reduce medical costs while enhancing patients’ quality of life. However, no studies to date have explored the combined clinical application of these two nursing methods, which is the focus of our study.

The evolution of national standards and their integration with nursing practices have increasingly shifted the focus toward nursing pathways aligned with these standards. This development combines nationally standardized nursing protocols with nursing pathways to create a coherent, interconnected, and progressive nursing model. Pathway-based TCM nursing interventions built on TCM clinical pathways standardize the care of single diseases, promote systematic intervention processes, highlight the efficacy and unique features of TCM, and make nursing operations more standardized. This enables nurses to provide planned and anticipatory care based on structured procedures. A preliminary retrospective analysis of clinical data from 4196 patients with diabetes revealed that integrating traditional Chinese nursing techniques with clinical treatments effectively alleviated patients’ discomfort. Nurses found this approach more practical and valuable in standardizing care, reducing variability, lowering costs, and improving care quality for patients with T2DM [8,9]. Nevertheless, no research has yet examined the simultaneous clinical application of these two nursing methods, which is the aim of our study.

Thus, we conducted a prospective, randomized, single-blind, parallel-controlled trial to further investigate the impact on patient outcomes of integrating nationally standardized nursing protocols with TCM nursing programs based on clinical pathways. These outcomes include hospitalization indicators (eg, length of stay and medical costs), self-management (health knowledge), clinical efficacy (blood glucose control, clinical symptoms, and quality of life), and patient satisfaction. We expect that the findings of this study will provide robust evidence for delivering personalized care, standardizing procedures, improving patients’ quality of life, and enhancing self-care and medication adherence after discharge.

## Methods

### Study Design

The intervention protocol for this study is titled “TCM Nursing Protocol for Type 2 Diabetes Based on Care Pathway.” The study uses a prospective, randomized, single-blind, parallel-controlled clinical design and aims to assess and demonstrate differences, supported by high-quality evidence, in outcomes between an intervention group and a control group that received standard care. The research team comprises nurses from the Department of Endocrinology at Guang’anmen Hospital, China Academy of Chinese Medical Sciences. The study participants are patients with T2DM admitted to our department between September 2022 and December 2024. The study is registered with the International Traditional Medicine Clinical Trial Registry (ITMCTR2022000048). Inclusion, exclusion, and elimination criteria are shown in Textbox 1.

**Inclusion, exclusion, and elimination criteria.**

**Inclusion criteria**

- The primary diagnosis must align with *International Classification of Diseases, Tenth Revision* codes for type 2 diabetes mellitus, specifically in the range of E11.2 to E11.9.
- Admission criteria must be met for inpatient treatment, as determined by the clinical physician.
- In cases where patients have concurrent diagnoses of other diseases, if these conditions do not require special treatment during the hospital stay and do not hinder the implementation of the clinical nursing pathway for the primary diagnosis, the patient can be included in that nursing pathway.
- Informed consent must be obtained from the patient and documented, indicating their understanding and agreement with the proposed care plan.

**Exclusion criteria**

- Patients who meet the diagnostic criteria for type 2 diabetes mellitus but are unable to comply with the treatment regimen.
- Patients with cognitive impairments or cognitive dysfunction.
- Patients with concurrent diagnoses of other diseases that require treatment during the hospitalization period.

**Elimination criteria**

- Patients whose condition worsens, resulting in a hospitalization duration exceeding 14 days and increased hospitalization costs.
- Patients with concomitant systemic diseases that require special treatment, leading to a hospitalization duration exceeding 14 days and increased costs.
- Patients with serious complications that arise during the course of treatment and necessitate discontinuation of the nursing pathway.
- Patient and family preferences and wishes that affect the implementation of this nursing pathway.

### Interventions

#### Intervention Plan

**Figure 1:**
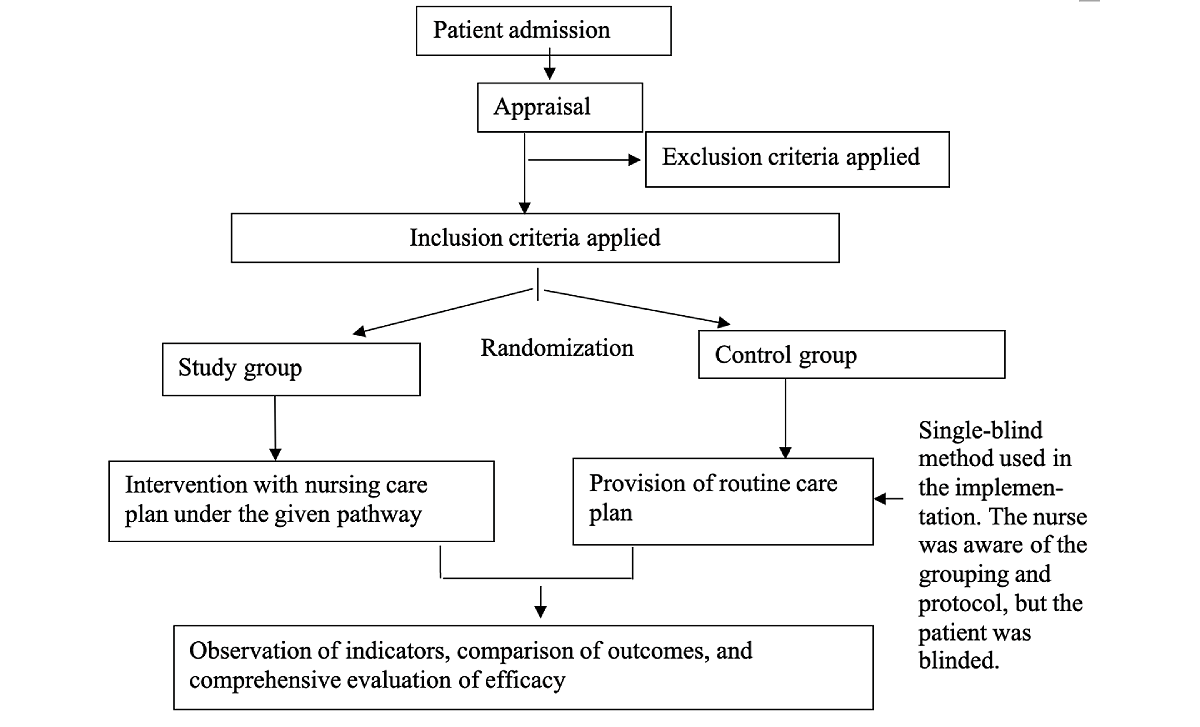
Study design and flow.

Upon admission, nurses provide admission education and assign eligible patients to the study group or the control group based on the time of admission (Figure 1). The intervention plans for both groups are outlined in a pathway form and include 3 components: executing medical orders, nursing tasks, and health education, along with TCM-specific treatments (Multimedia Appendix 1). For the control group, nurses subjectively implement the nursing plan according to the pathway form. The study group strictly follows the TCM nursing pathway for T2DM.

#### Implementation of the TCM Nursing Pathway for T2DM

Upon admission, nurses provide admission education and assess the patient’s condition using an evaluation form for the TCM nursing pathway for T2DM. This assessment determines whether the patient qualifies for inclusion in the study. Eligible patients receive the TCM nursing pathway for T2DM. For patients included in the study, nurses ensure that physicians perform a thorough examination upon admission. The TCM nursing pathway form for T2DM is distributed, and patients are informed about their treatment and the nursing plan during hospitalization. This ensures patients understand the medical and nursing measures to be implemented.

During hospitalization, the responsible nurse uses the evaluation form of the TCM nursing pathway for T2DM to develop an appropriate care plan. This plan may include TCM techniques, syndrome differentiation–based dietary therapy, exercise and health practices, herbal prescriptions, and emotional regulation. The study group strictly adheres to the schedule outlined in the TCM nursing pathway for T2DM. Nurses coordinate with relevant departments promptly to complete necessary examinations. The control group receives routine care.

If there are any changes in the patient’s condition, the responsible nurse promptly analyzes and addresses the changes, adjusting the sequence of items in the TCM nursing pathway as necessary. The head nurse checks the implementation of the TCM nursing pathway for T2DM and the routine care plan daily. The head nurse ensures compliance with the nursing pathway protocol and evaluates the quality of care promptly. Relevant data are collected and evaluated at the time of the patient’s discharge.

#### Sample Size Calculation

A literature-based approach was used to compare the preset effect, with α=.05 and β=.10, against the known efficacy of general nursing from previous studies. Based on methods found in the literature [10] and conclusions from earlier research, the historical effective rate (very satisfied + satisfied) was determined to be approximately 74.5% [9]. The effective rate for this pathway nursing program is projected to be 85%. Using *Z* values from standard tables (*Z*1 – α / 2 = 1.96; *Z*1 – β = 1.28), the sample size for each group was estimated as follows:

N1 = N2 = (*Z*1 – α / 2 + *Z*1 – β) 2 [P1(1 – P1) + P2(1 – P2)] / δ2 = (1.96 + 1.28) 2 [0.745 × (1 – 0.745) + 0.85 × (1 – 0.85)] / (0.85 – 0.745) 2 = 296. Thus, 242,272 indicated 297 cases, and the sample size of this study was 297 × 2 = 594 cases.

#### Randomization Method

The randomization method used in this study involves selecting an appropriate block size and assigning participants into 2 groups (the study group and the control group) in a 1:1 ratio. Using the *PROC PLAN* procedure statement in SAS (version 9.2; SAS Institute) and specifying a seed number, a randomized allocation was generated for 594 participants. This process produced a complete randomization table, listing the treatment assignments corresponding to sequential serial numbers from 001 to 594.

#### Blinding Method

This is a single-blind study, and patients with diabetes are unaware of their group assignment.

#### Outcomes

Researchers use standardized questionnaires to collect baseline data. The primary efficacy indicator is a quality of life scale (the Diabetes-Specific Quality of Life Scale) [11]. Secondary efficacy indicators include the length of hospital stay, medical costs, blood glucose control, patient symptom scores, patient satisfaction, and the level of health knowledge acquired at admission. Data are collected 1 day before discharge and at 4 weeks, 12 weeks, and 24 weeks after discharge.

#### Harm

Apart from the risk of data violations, there is no anticipated harm to the study participants. The investigator will be identified by experimenter number to reduce the risk. Although greater adherence to treatment is expected in the intervention group, no systematic differences in diabetes management are expected.

### Ethical Considerations

The ethical principles guiding this study include the Measures for Ethical Review of Biomedical Research Involving Humans, Good Clinical Practice for Drug Trials, Regulations on Clinical Trials of Medical Devices, Ethical Review Guidance for Drug Clinical Trials, Clinical Ethical Review Management Guidelines for Traditional Chinese Medicine Hospitals, the Declaration of Helsinki, and the International Ethical Guidelines for Biomedical Research Involving Human Subjects.

The trial has been approved by the Ethics Committee of Guang’anmen Hospital, China Academy of Chinese Medical Sciences (2022-022-KY-01). Prior to the implementation of the study, informed consent must be obtained from patients. Participation in this study is entirely voluntary. Patients may choose not to participate or withdraw from the study at any time without providing a reason, and participation requires signing an informed consent form.

All patient data collected during the trial will remain confidential. Medical records will be identified using research ID numbers instead of names. Identifiable information will not be disclosed to anyone outside the research team unless the patient provides explicit permission.

In accordance with relevant national regulations, in the event of research-related injuries, the study institution, Guang’anmen Hospital of the China Academy of Chinese Medical Sciences, will bear the corresponding medical costs and provide appropriate financial compensation.

For any questions regarding participant rights in this study, individuals can contact the Ethics Committee of Guang’anmen Hospital, China Academy of Chinese Medical Sciences.

The study form will be kept by the data collector in a folder in the study office. Study data will be processed without participants being recognizable. Only the researcher responsible for the analysis can access the final data set; after the trial study is published, we will provide research references and the scientific basis for relevant clinical practice, formulate the implementation specifications of the nursing program under the chosen pathway, and promote the research results in the industry.

### Statistical Analysis

We will use SPSS (version 26.0; IBM Corp) to perform the statistical analysis. Descriptive statistics for quantitative variables will include at least the mean and SD or the median and IQR. Descriptive statistics for qualitative variables will include at least the frequency and percentage for each category. Comparisons between the 2 groups will be conducted using an independent-sample *t* test (for normally distributed data with equal variances) or the nonparametric rank-sum test. For unordered categorical data, comparisons between the 2 groups will be made using the *χ*^2^ test or Fisher exact test, while ordinal categorical data will be analyzed using nonparametric tests. All statistical tests will be 2-sided, with a *P*<.05 considered statistically significant.

## Results

This trial received funding in August 2021 and is currently ongoing. The first patient was enrolled in September 2022, and the study is expected to conclude in December 2024. The trial uses a single-blind design where nurses are aware of group assignments and intervention details, but the patients remain blinded. Final results are planned for publication in the first quarter of 2025.

To date, 380 patients have been recruited, with 172 excluded, resulting in 202 randomized participants (101 in the study group and 101 in the control group). Details on the screening process are provided as a Consolidated Standards of Reporting Trials (CONSORT) flowchart in Multimedia Appendix 2.

This study primarily aims to compare health knowledge, comprehension, and scores between the 2 groups, with the goal of optimizing an innovative model for the TCM nursing pathway for T2DM. The findings are expected to enhance the standardization and precision of clinical nursing practices. The pathway will be validated clinically, combining theory with practice through prospective research to provide high-level clinical evidence. This will contribute to the development of a high-quality TCM nursing pathway, expand the global influence of TCM, and support national economic growth and public health.

## Discussion

### Overview

Data show a significant increase in China in the incidence of diabetes over 30 years, leading to a high prevalence of acute and chronic complications. Simultaneously, deficiencies in patient awareness, understanding, treatment rates, and control rates have become evident in diabetes care. This underscores the urgent need to explore and improve diabetes care methods.

In recent years, China’s economic growth has created opportunities for more targeted and standardized care, providing promising prospects for development. Under the framework of National Standards + Nursing Pathways, TCM-based nursing models have become prominent in diabetes clinical care. However, despite positive outcomes with existing standards, a comprehensive clinical pathway management model has yet to be established, resulting in inconsistencies and nonstandardized practices.

Thus, it is crucial to conduct in-depth research to integrate these methods and improve the effectiveness of nursing practices. Exploring and developing nursing plans that combine clinical pathways with TCM principles is essential. High-quality prospective clinical research is needed to meet patients’ increasing demand for improved health care quality and to refine standardized nursing protocols.

Since the Ministry of Health launched the clinical pathway pilot project in 2009, the effectiveness of clinical pathway management in improving health care quality and controlling medical costs has become increasingly evident. Against the backdrop of advocating “high quality, high efficiency, and low cost,” studying the application of clinical nursing pathways in China’s health care quality management is of significant importance. This emphasizes the need for further development and research into the implementation of a TCM nursing model for T2DM based on National Standards + Nursing Pathways.

### Principal Findings

This study focuses on comparing health knowledge and scores in the 2 groups, aiming to optimize an innovative model for the TCM nursing pathway for T2DM. This approach should help enhance the standardization and precision of clinical nursing practices. Its application in the clinic will combine theory with practice, providing high-level clinical evidence through prospective research. The goal is to establish a high-quality TCM nursing pathway that will expand the international influence of TCM and support economic and public health development.

### Strengths and Limitations

Building on prospective findings from previous studies, this study will use a prospective design to enhance the level and reliability of evidence. The study’s significance also lies in providing a foundation for high-quality clinical evaluation that will enable selecting targeted and distinctive TCM clinical nursing pathways. The aim is to improve the clinical application and quality of standard nursing procedures within nursing projects based on a T2DM nursing pathway.

However, this study uses a single-center design and does not include additional hospitals, which is a limitation. Nevertheless, the focus is on optimizing the TCM nursing pathway at this center and obtaining high-quality evidence of its effectiveness to facilitate broader future applications and clinical use.

### Future Directions

The TCM nursing pathway explored in this study has a solid foundation, demonstrating its potential for effective care. We anticipate that as the study progresses, high-quality evidence will confirm its efficacy. Subsequently, this TCM nursing pathway will be promoted in more hospitals, benefiting a larger population of patients with diabetes.

### Conclusions

This study aims to integrate existing national standardized nursing protocols with clinical nursing pathways to implement more efficient and higher-quality clinical nursing practices. It seeks to standardize nursing procedures, improve patients’ quality of life, and promote self-care and medication adherence after discharge.

## Appendix

Multimedia Appendix 1 Clinical pathway form.

Multimedia Appendix 2 Consolidated Standards of Reporting Trials (CONSORT) flowchart of participant recruitment and allocation to date.

## Abbreviations

CONSORT: Consolidated Standards of Reporting Trials
TCM: traditional Chinese medicine
T2DM: type 2 diabetes mellitus
