# Supplementary material for: Evaluation of the Clinical Nursing Effects of a Traditional Chinese Medicine Nursing Program Based on Care Pathways for Patients With Type 2 Diabetes: Protocol for a Randomized Controlled Clinical Trial
Source: JMIR Res Protoc. 2025 Mar 31;14:e58951. doi: 10.2196/58951 (PMC11997517; doi:10.2196/58951)
Supplement: Multimedia Appendix 1 [file resprot_v14i1e58951_app1.doc]

**Subject ID: / / / / /**

**Subject name abbreviation / / / / /**

**Clinical Pathway Form for the Study on the Clinical Application of TCM Nursing Plans for Type 2 Diabetes Mellitus (Xiaoke Syndrome)**

| Time (Days) | Nursing Pathway | | |
| --- | --- | --- | --- |
|  | Medical Orders | Nursing Tasks | Health education and traditional Chinese medicine characteristic treatment |
| 1 | Long-term Orders:  1. Implement graded nursing care.  2. Type 2 diabetes diet.  3. Diabetes medication. Non-pharmacological therapy.  Temporary Orders:  1. Blood glucose, blood routine, urine routine.  2. ECG, chest X-ray, etc.  3. Intravenous fluids (if necessary).  4. Islet function tests.  5. Symptom-specific treatment (hypoglycemia, glucose reduction, ketone correction, etc.).  6. Advise patient to fast after midnight; draw blood in the morning.  7. Transfer patients with acute complications to the corresponding pathway. | Admission nursing:  Monitor vital signs  Complete nursing record form  Assist in completing immediate urine and electrocardiogram examinations  Individuals with safety hazards should follow medical advice to arrange a companion bed  Admission assessment:  Skin, tubing, hygiene  Knowledge of diabetes  Complete assessment of living ability, falls and falls from bed  Complete diabetes specialist physical examination  Dialectical classification  Main certificate:  Liver and stomach stagnation heat Gastrointestinal solid heat  Spleen deficiency and stomach heat Upper heat and lower cold  Deficiency of Yin and Excessive Fire Deficiency of Qi and Yin  Yin Yang deficiency  Other:  Certificate:  Blood stasis syndrome, phlegm syndrome, dampness syndrome, turbidity syndrome  Clinical care (traditional Chinese medicine characteristic treatment):  Tip: Acupoint increase or decrease, if not implemented, please indicate the horizontal line  1. Increased urine output:  Acupoint massage  Moxibustion  Chinese herbal hot ironing and compress  Microcirculation therapy  Other:  2. Drink more when your mouth is dry  Ear acupressure  Acupoint tapping  Moxibustion  Chinese herbal hot ironing and compress  Microcirculation therapy  Other:  3. Eating too much can lead to hunger  Ear acupressure  Acupoint tapping  Moxibustion  Chinese herbal hot ironing and compress  Microcirculation therapy  Other:  4. Fatigue and fatigue  Moxibustion  Acupoint application  Acupoint tapping  Chinese herbal hot ironing and compress  Microcirculation therapy  Soak and wash traditional Chinese medicine  Other:  5. Limb numbness, pain, and coldness  Moxibustion  Soak and wash traditional Chinese medicine  Acupoint application  Acupoint massage  Ear acupressure  Chinese herbal hot ironing and compress  Microcirculation therapy  Other:  6. Blurred vision  Acupoint massage  Traditional Chinese Medicine Nebulization  Ear acupressure  Microcirculation therapy  Traditional Chinese Medicine Pillow  Other:  7. Itching skin  Ear acupressure  Microcirculation therapy  Other:  8. Weakness and soreness in the waist and knees  Eight section brocade  Ear acupressure  Acupoint massage  Moxibustion  Chinese herbal hot ironing and compress  Microcirculation therapy  Soak and wash traditional Chinese medicine  Chinese herbal retention enema  Other:  9. Constipation  Abdominal massage  Ear acupressure  Acupoint massage  Moxibustion  Chinese herbal hot ironing and compress  Acupoint application  Other:  High quality care:  Provide life assistance  Do a good job of six cleanliness | Individualized health education  Introduce the bed management doctor and responsible nurse  Complete admission education  Explain the relevant knowledge of hypoglycemia  Develop a clinical nursing plan based on nursing evaluation  Distribute personalized health education manuals  Develop a dialectical diet plan based on dietary habits  Explain medication methods and precautions  Daily life guidance  Introduce the precautions before each inspection  Individuals with pancreatic islet function should be informed and prepared  Explain relevant knowledge to hypoglycemic/ketogenic personnel  Fasting and drinking after 10 pm, blood samples will be taken and fasting B-ultrasound will be performed the next morning |
| Execution time: Performer: | | | |
| Mutation: Yes  No   reason: | | | |

| Time (Days) | Nursing Pathway | | |
| --- | --- | --- | --- |
|  | Medical Orders | Nursing work | Health education and traditional Chinese medicine characteristic treatment |
| 2～6 | Long term medical advice  1. Implement graded nursing care  Type 2 diabetes diet  3. Medication for diabetes  4. Non pharmacological therapies  STAT order  1. Internal and external examinations  2. diabetes related examination  3. Follow medical advice and implement traditional Chinese medicine nursing techniques  4. Follow medical advice to handle changes in the condition  5. Patients with acute complications (transferred to the corresponding nursing pathway) | □ Routine nursing care for type 2 diabetes  Reasonably arrange the sequence of multiple inspections  Arrange family members or field personnel to accompany those with safety hazards  □ Arrange them to complete the examination related to diabetes complications in time  □ Check the results of various examinations and pay attention to the examinations related to diabetes complications  Complete nursing records on time  □ Conduct regular house patrols and monitor changes in the patient's condition  Clinical care (traditional Chinese medicine characteristic treatment):  Tip: Acupoint increase or decrease, if not implemented, please indicate the horizontal line  1. Increased urine output:  Acupoint massage  □ Moxibustion  □ Chinese herbal hot ironing and compress  Microcirculation therapy  Other:  2. Drink more when your mouth is dry  Ear acupressure  □ Acupoint tapping  □ Moxibustion  □ Chinese herbal hot ironing and compress  Microcirculation therapy  Other:  3. Eating too much can lead to hunger  Ear acupressure  □ Acupoint tapping  □ Moxibustion  □ Chinese herbal hot ironing and compress  Microcirculation therapy  Other:  4. Fatigue and fatigue  □ Moxibustion  Acupoint application  □ Acupoint tapping  □ Chinese herbal hot ironing and compress  Microcirculation therapy  Soak and wash traditional Chinese medicine  Other:  5. Limb numbness, pain, and coldness  □ Moxibustion  Soak and wash traditional Chinese medicine  Acupoint application  Acupoint massage  Ear acupressure  □ Chinese herbal hot ironing and compress  Microcirculation therapy  Other:  6. Blurred vision  Acupoint massage  □ Traditional Chinese Medicine Nebulization  Ear acupressure  Microcirculation therapy  □ Traditional Chinese Medicine Pillow  Other:  7. Itching skin  Ear acupressure  Microcirculation therapy  Other:  8. Weakness and soreness in the waist and knees  □ Eight section brocade  Ear acupressure  Acupoint massage  □ Moxibustion  □ Chinese herbal hot ironing and compress  Microcirculation therapy  Soak and wash traditional Chinese medicine  □ Chinese herbal retention enema  Other:  9. Constipation  □ Abdominal massage  Ear acupressure  Acupoint massage  □ Moxibustion  □ Chinese herbal hot ironing and compress  Acupoint application  Other:  High quality care:  Provide life assistance  □ Do a good job of six cleanliness (hair, nails, skin, perineum, mouth, bed unit) | Assist patients in ordering meals and correcting unhealthy eating habits based on the dialectical diet plan  Develop an exercise and health plan based on the condition and blood sugar fluctuations  Knowledge related to insulin injection  □ Medication methods and precautions  Notify and explain the addition or modification of drugs  Explain the precautions for non pharmacological therapies  □ Relevant knowledge of diabetes foot  □ Relevant knowledge of diabetes eye use  Knowledge related to blood glucose monitoring  □ Emotional regulation  □ Clinical care related content  Self care and precautions for traditional Chinese medicine characteristic treatment  □ Safety education for outdoor inspections  Provide targeted guidance based on the results of various inspections, tests, and consultations  □ Educate about the results of examinations related to diabetes complications  Provide targeted guidance for changes in the patient's condition  □ Educational loopholes |
| Execution time: Performer: | | | |
| Mutation: Yes  No   reason: | | | |

| Time (Days) | Nursing pathway | | |
| --- | --- | --- | --- |
|  | Execute medical orders | Nursing work | Health education and traditional Chinese medicine characteristic treatment |
| 7-10  (Discharge) | Long term medical advice  1. Implement graded nursing care  Type 2 diabetes diet  3. Medication for diabetes  4. Non pharmacological therapies  STAT order  1. Internal and external examinations  2. diabetes related examination  3. Follow medical advice and implement traditional Chinese medicine nursing techniques  4. Follow medical advice to handle changes in the condition  discharge order  1. Discharge with medication  2. Stop various medical orders  3. Patients with acute complications (transferred to the corresponding nursing pathway) | □ Routine nursing care for type 2 diabetes  Reasonably arrange the sequence of multiple inspections  Arrange family members or field personnel to accompany those with safety hazards  □ Arrange them to complete the examination related to diabetes complications in time  □ Check the results of various examinations and pay attention to the examinations related to diabetes complications  Complete nursing records on time  □ Conduct regular house patrols and monitor changes in the patient's condition  Clinical care (traditional Chinese medicine characteristic treatment):  Tip: Acupoint increase or decrease, if not implemented, please indicate the horizontal line  1. Increased urine output:  Acupoint massage  □ Moxibustion  □ Chinese herbal hot ironing and compress  Microcirculation therapy  Other:  2. Drink more when your mouth is dry  Ear acupressure  □ Acupoint tapping  □ Moxibustion  □ Chinese herbal hot ironing and compress  Microcirculation therapy  Other:  3. Eating too much can lead to hunger  Ear acupressure  □ Acupoint tapping  □ Moxibustion  □ Chinese herbal hot ironing and compress  Microcirculation therapy  Other:  4. Fatigue and fatigue  □ Moxibustion  Acupoint application  □ Acupoint tapping  □ Chinese herbal hot ironing and compress  Microcirculation therapy  Soak and wash traditional Chinese medicine  Other:  5. Limb numbness, pain, and coldness  □ Moxibustion  Soak and wash traditional Chinese medicine  Acupoint application  Acupoint massage  Ear acupressure  □ Chinese herbal hot ironing and compress  Microcirculation therapy  Other:  6. Blurred vision  Acupoint massage  □ Traditional Chinese Medicine Nebulization  Ear acupressure  Microcirculation therapy  □ Traditional Chinese Medicine Pillow  Other:  7. Itching skin  Ear acupressure  Microcirculation therapy  Other:  8. Weakness and soreness in the waist and knees  □ Eight section brocade  Ear acupressure  Acupoint massage  □ Moxibustion  □ Chinese herbal hot ironing and compress  Microcirculation therapy  Soak and wash traditional Chinese medicine  □ Chinese herbal retention enema  Other:  9. Constipation  □ Abdominal massage  Ear acupressure  Acupoint massage  □ Moxibustion  □ Chinese herbal hot ironing and compress  Acupoint application  Other:  High quality care:  Provide life assistance  □ Do a good job of six cleanliness (hair, nails, skin, perineum, mouth, bed unit)  discharge nursing  Complete the writing of the final nursing record and organize the medical records  Individualized health education manual for patients to take away  Evaluation of Compliance and Satisfaction with Traditional Chinese Medicine Featured Treatment  1. Abdominal massage  □ Compliance □ Partial compliance □ Non compliance  □ Satisfied □ Fair □ Unsatisfied  2. Ear acupressure  □ Compliance □ Partial compliance □ Non compliance  □ Satisfied □ Fair □ Unsatisfied  3. Acupoint application  □ Compliance □ Partial compliance □ Non compliance  □ Satisfied □ Fair □ Unsatisfied  4. Acupoint massage  □ Compliance □ Partial compliance □ Non compliance  □ Satisfied □ Fair □ Unsatisfied  5. Moxibustion  □ Compliance □ Partial compliance □ Non compliance  □ Satisfied □ Fair □ Unsatisfied  6. Chinese herbal hot ironing and compress  □ Compliance □ Partial compliance □ Non compliance  □ Satisfied □ Fair □ Unsatisfied  7. Traditional Chinese Medicine Retention Enema  □ Compliance □ Partial compliance □ Non compliance  □ Satisfied □ Fair □ Unsatisfied  8. Acupoint tapping  □ Compliance □ Partial compliance □ Non compliance  □ Satisfied □ Fair □ Unsatisfied  9. Chinese medicine soaking and washing  □ Compliance □ Partial compliance □ Non compliance  □ Satisfied □ Fair □ Unsatisfied  Responsible for evaluating the use of the plan  □ Strong practicality  □ Strong practicality  □ General practicality  □ Not practical | Provide special dietary guidance based on inspection and testing reports  Adjust the exercise plan according to specific circumstances  Correction of insulin injection errors in patients  Notify and explain the addition or modification of drugs  □ Knowledge of diabetes foot and eye use in patients with defect detection and leak repair  □ Teach patients to make their own diabetes patient cards  Provide targeted guidance based on the results of various inspections, tests, and consultations  Provide targeted guidance for changes in the patient's condition  □ Educational loopholes  Health knowledge Q&A  Discharge education  □ Handling of discharge procedures  □ Discharge medication guidance |
| Execution time: Performer: | | | |
| Mutation: Yes  No   reason: | | | |
