# Supplementary figures and images for: Evaluation of the Clinical Nursing Effects of a Traditional Chinese Medicine Nursing Program Based on Care Pathways for Patients With Type 2 Diabetes: Protocol for a Randomized Controlled Clinical Trial
Source: JMIR Res Protoc. 2025 Mar 31;14:e58951. doi: 10.2196/58951 (PMC11997517; doi:10.2196/58951)

Enrollment

Allocation

Follow-Up

Analysis

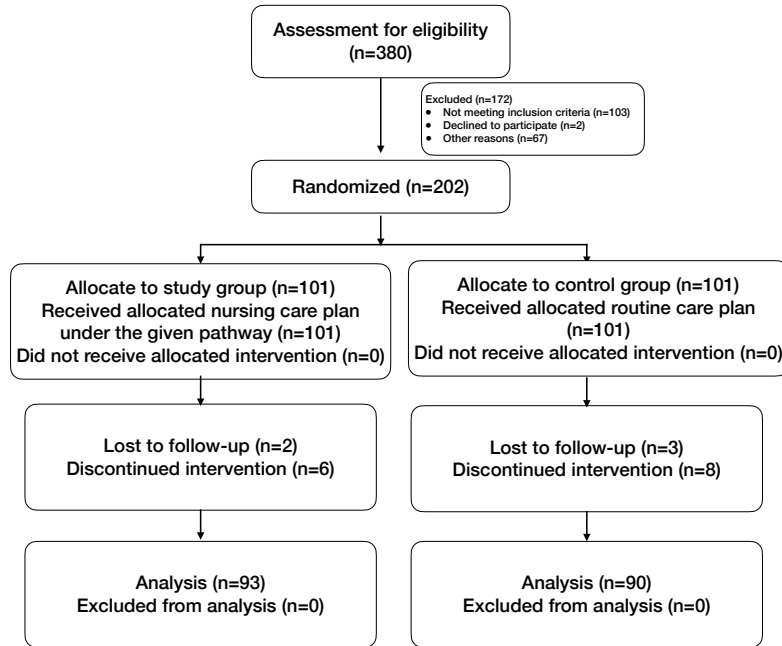

Supplement: Multimedia Appendix 2 [file resprot_v14i1e58951_app2.pdf]
